# Motivational Interviewing in Childhood Obesity Treatment

**DOI:** 10.3389/fpsyg.2015.01732

**Published:** 2015-11-12

**Authors:** Maria Borrello, Giada Pietrabissa, Martina Ceccarini, Gian M. Manzoni, Gianluca Castelnuovo

**Affiliations:** ^1^Department of Psychology, University of BergamoBergamo, Italy; ^2^Psychology Research Laboratory, IRCCS Istituto Auxologico Italiano, Ospedale San GiuseppePiancavallo, Italy; ^3^Department of Psychology, Catholic University of MilanMilan, Italy; ^4^Faculty of Psychology, eCampus UniversityNovedrate, Italy

**Keywords:** motivational interviewing, childhood, obesity, review, children, overweight, pediatric obesity

## Abstract

Obesity is one of today’s most diffused and severe public health problems worldwide. It affects both adults and children with critical physical, social, and psychological consequences. The aim of this review is to appraise the studies that investigated the effects of motivational interviewing techniques in treating overweight and obese children. The electronic databases PubMed and PsychINFO were searched for articles meeting inclusion criteria. The review included studies based on the application of motivational interviewing (MI) components and having the objective of changing body mass index (BMI) in overweight or obese children from age 2 to age 11. Six articles have been selected and included in this review. Three studies reported that MI had a statistically significant positive effect on BMI and on secondary obesity-related behavior outcomes. MI can be applicable in the treatment of overweight and obese children, but its efficacy cannot be proved given the lack of studies carried out on this specific sample.

## Introduction

Obesity is a complex disorder that involves an excessive amount of body fat, generally caused by the lack of balance between energy intake and energy expenditure (Bray and Popkin, 1998).

Overweight and obesity is measured referring to the BMI, defined as the weight in kilograms divided by the square of the height in meters (kg/m^2^) (Pietrabissa et al., 2012). This index is a widely used way to classify overweight and obesity for both adults and children, but the results are interpreted differently. For children and adolescents between 2 and 20 years-old, BMI is interpreted relative to a child’s age and gender, because the amount of body fat changes with age and varies by gender (American Academy of Pediatrics Committee on Nutrition, 2003; Flegal and Ogden, 2011). In addition, the accuracy of BMI varies substantially according to the individual child’s degree of body fatness. Thus, percentiles specific to age and gender classify underweight, healthy weight, overweight, and obesity in children. Overweight condition is determined when BMI is comprised between the 85th and 95th percentile for gender and age. A child is identified as obese when his BMI is equal or above the 95th percentile (Barlow, 2007).

Worldwide childhood obesity rates increased substantially from 1990 to 2010, with an increase of 60% children suffering from obesity both in developing and developed countries; such rates are expected to keep growing all around the world and reach a total number of about 60 million overweight and obese preschool children in 2020 (De Onis et al., 2010).

The obesity widespread among the young people is generating significant social concern for public health, not only for the number of children affected but also because of its consequences. Overweight condition in children is likely to be maintained in adulthood (Singh et al., 2008), influencing and affecting the young for their entire life because of the development of important health problems, such as cardiovascular, metabolic, pulmonary, gastrointestinal, and orthopedic (Daniels, 2009). Studies on childhood obesity also report psychological and social consequences (Bean et al., 2008). Main psychological impairments are ADHD, poor health-related quality of life (Pulgaron, 2013), low self-esteem related to loneliness, sadness, and nervousness (Strauss, 2000) and a negative self-image from the age of 5 years (Davison and Birch, 2001). Also, in relationship with peers obese children are often labeled as unhealthy, inadequate at school and lazy (Hill and Silver, 1995).

Given the dimension of the problem, treatments of childhood obesity are recognized as of extreme importance. Fundamental is to produce a change of food intake and quality (Latzer et al., 2009), to increase their effort in physical activity and to reduce sedentary habits (Epstein et al., 1995, 2000) Parents are very important for any success in treatment, both helping children introducing and managing new healthy living and eating habits and representing themselves a role model (Campbell and Crawford, 2001). Because of the pivotal role of families’ participation in weight control programs, it is then necessary to support and work with parents to change children unhealthy habits (Golan and Crow, 2004).

### Motivational Interviewing

The use of MI has been reported as a promising approach in the treatment of obesity (Resnicow et al., 2006). MI is defined as “a collaborative, goal-oriented style of communication with particular attention to the language of change. It is designed to strengthen personal motivation for and commitment to a specific goal by eliciting and exploring the person’s own reasons for change within an atmosphere of acceptance and compassion” (Miller and Rollnick, 2013). It can help the change of family lifestyles and eating habits that is crucial to succeed in treating overweight or obesity children condition. MI practitioner stimulates the family to express their own reasons for and against change problematic conducts and to reflect on the inconsistencies or gap between future goals and current behavior (Kirk et al., 2005).

Despite conventional more directive approaches based on clinicians’ prescriptions and on confrontation, MI is centered on supporting the client’s autonomy and collaboration as well as on evocating her/his motivation to change making the person actively taking part in the caring and changing process. The counselor does not establish any goal for the client nor imposes his/her own point of view but, through the use of a “guiding style”, the professional helps the client developing both new coping strategies and future plan of action (Rollnick et al., 2008; Rollnick et al., 2010).

Following the four processes (engaging, focusing, evoking, and planning), practitioner leads the patient to autonomously individuate and choose the best way to proceed in change (Miller and Rollnick, 2013).

MI has been used with good results in different health domains such as substance abuse, smoking, diabetes, and weight loss (e.g., Britt et al., 2004; Hettema et al., 2005; Rubak et al., 2005; Martins and McNeil, 2009; Lundahl et al., 2013).

The majority of studies investigating the use of MI in reducing weight among overweight an obese people, typically consider adult populations (e.g., Carels et al., 2007; Van Dorsten, 2007; West et al., 2007; Armstrong et al., 2011; Hardcastle et al., 2013), and their promising results support the efficacy of MI in enhancing motivation for lifestyle change (Pietrabissa et al., 2013; Castelnuovo et al., 2014). Instead, literature on the application of MI with adolescents is poor (e.g., Berg-Smith et al., 1999; Resnicow et al., 2005; Neumark-Sztainer et al., 2010; Macdonell et al., 2012; Bean et al., 2015) and even fewer are the studies focused on overweight or obese children. Outcomes reveal a positive effect of MI on both adolescents’ improvement on healthier eating behavior and physical activity as well as a perceived high satisfaction of the motivational sessions among the clients.

In order to maximize its efficacy, MI is flexibly adapted to characteristics and needs of the patient (Emmons and Rollnick, 2001) and, especially for children, the age of the single client is considered (Resnicow et al., 2006).

This review focuses on studies investigating the application of MI for the treatment of obese children up to 11 years-old and, differently from previous ones (Resnicow et al., 2006; Limbers et al., 2008; Martins and McNeil, 2009; Christie and Channon, 2014), does not consider works that involve adolescents. The aim of this contribution is to appraise the studies that investigated the effects of MI techniques in treating overweight and obese children.

## Methods

### Study Selection

A broad literature search has been conducted in July 2014 to identify studies based on interventions with a MI component to change BMI in the treatment of overweight or obese children aged 2–11 years-old.

Two electronic databases were searched, PubMed and PsychINFO, using the following keywords: “motivational interviewing”, “childhood obesity”, “children overweight”, “pediatric obesity”.

Additional inclusion criteria were: publication between 2007 and June 2014, inclusion of overweight (BMI at or above the 85th percentile and lower than the 95th percentile for children of the same age and sex) or obese (BMI at or above the 95th percentile for children of the same age and sex) children, experimental, or quasi-experimental design, articles written in the English language.

Studies that included parents were considered only if the primary target of the motivational intervention was the child.

## Results

### Description of Selected Studies

Six articles were finally included.

An overview of participants’ characteristics of each included study is provided in **Table 1**. The intervention type and main results are shown in **Table 2**.

**Table 1:** Selected article characteristics of the sample.

Article	*N*	Sample age	Sample weight
Schwartz et al., 2007	91	3–7	85th percentile ≤ BMI ≤ 95th percentile or 50th percentile ≤ BMI ≤ 85th percentile with at least 1 parent’s BMI ≥ 30
Taveras et al., 2011	475	2–6	BMI ≥ 95th percentile or 85^th^ percentile ≤ BMI ≤ 95^th^ percentile if at least one parent was overweight
Davoli et al., 2013	372	4–7	85th percentile ≤ BMI ≤ 95th percentile
Small et al., 2013	60	4–8	Overweight or obese children according to BMI percentile
van Grieken et al., 2013	637	5	Overweight not obese children
Wong and Cheng, 2013	185	9–11	Obese children according to the Hong Kong Growth Survey (HKGS) sex specific reference charts of medium weight-for-height

**Table 2:** Selected article intervention description and main results.

Study	Design	Objective	Outcomes	Comparison groups (N)	Intervention description	Training MI	Results
Schwartz et al., 2007	NRS	To implement an office-based obesity prevention program using MI.	Change in the BMI for age percentile.	(1) Control (21) (2) Minimal intervention (40) (3) Intensive intervention (30)	Minimal intervention group received one MI session for 10–15 min. Intensive intervention group received 2 MI sessions, one of 10–15 min duration and of 45–50 min long.	2 days session before the intervention. Audiotapes for clinical supervision with telephone feedback and coaching.	Decrease of BMI percentiles in the control (0.6), minimal (1.9), and intensive (2.6) groups. BMI differences between the three groups were non-significant (*p* = 0.85).
Taveras et al., 2011	RCT	To examine the effectiveness of a primary care-based obesity intervention.	Change in BMI and obesity-related behaviors.	(1) usual care (204) (2) intervention (271)	Use MI for four sessions in person for 25 min and three telephone calls for 15 min.	Pediatric nurse practitioners trained in MI.	Intervention participants had a smaller, non-significant increase in BMI (-0.21 kg/m^2^; *p* = 0.15), greater decreases in TV viewing (-0.36 h/day; *p* = 0.01), had slightly greater decreases in fast food (-0.16 servings/week; *p* = 0.07) and sugar sweetened beverages (-0.22 servings/day; *p* = 0.15). Significant effects observed on BMI among females (-0.38 kg/m^2^; *p* = 0.03) and among participants in households with annual incomes $50,000 or less (-0.93 kg/m^2^; *p* = 0.01).
Davoli et al., 2013	RCT	To evaluate the effect of family pediatrician–led motivational interviews (MIs) on BMI of overweight children.	BMI score variation, percentage of positive changes in parent-reported dietary behaviors and in physical activity.	(1) usual care (185) (2) intervention (187)	Five MI meetings based on the Transtheoretical model.	20-h training course on MI conducted by specialized psychologists.	There was a significant difference in BMI between intervention and control groups (difference = -0.30, *p* = 0.007). MI had effect in children whose mother had a high educational level (-1.04 kg/m^2^; *p* = 0.008) and in girl (Transtheoretical0.51 kg/m^2;^ *p* = 0.072). Positive changes in parent-reported lifestyle behaviors occurred more frequently in the MI group.
Small et al., 2013	RCT	To determine the feasibility and preliminary effects of a theoretically based, primary care intervention on the physical outcomes.	BMI percentile, waist circumferences, waist by height ratio.	(1) control group (33) (2) treatment (34)	Four brief MI sessions.	Not specified.	In treatment group reduced waist circumference and waist-by-height ratio immediately after the intervention that persisted for 3 (*f* = 0.33) and 6 months (*f* = 0.35). BMI and BMI percentile were not differentially affected.
van Grieken et al., 2013	RCT	To assess the effectiveness of a prevention protocol among 5-year-old overweight children.	BMI and waist circumference after 2 years.	(1) usual care (349) (2) intervention (288)	Three structured lifestyle counseling sessions using a MI approach, if needed.	1-day workshop on MI.	There was no overall difference between intervention and control condition. Mildly overweight children (baseline BMI 17.25 and 17.50) in the intervention condition showed a significantly smaller increase in BMI at follow-up (estimated adjusted mean difference -0.67, *p* = 0.024 and -0.52, *p* = 0.045, respectively). compared to the control condition.
Wong and Cheng, 2013	Pre-post quasi-experimental study	To assess the effects of motivational interviewing for obese children and telephone consultation for parents to promote weight loss in obese children.	Change in weight for-height percentage, in weight-related behaviors (calorie intake and calorie consumption) and weight-related anthropometric measures (BH, BW, BMI, Fat%, TSF, Abd, WC, HC,SBP, DBP)	(1) control (49) (2) MI (70) (3) MI+ (66)	Children in MI group received five MI sessions. Children in the MI+ group received five MI sessions and five telephone consultation calls for their parents.	Training in MI skills.	Children in both the MI and MI+ groups showed significant improvement in their weight-related behaviors and obesity-related anthropometric measures. Significant differences have been found in pre-and post- intervention for MI group: e.g., calories intake decrease (mean difference 389.57; *p* < 0.01); calories consume in PA exercise increase (mean difference 2052.10; *p* < 0.01); MWH (*t* = 8.67, *p* < 0.01), BMI (*t* = 5.36, *p* < 0.01), Fat% (*t* = 7.07, *p* < 0.01), WC (*t* = 9.92, *p* < 0.01). Significant differences have been found in pre-and post- intervention for MI+ group: e.g., calories intake decrease (mean difference 376.65; *p* < 0.01); calories consume in PA exercise decrease (mean difference 2590.64; *p* < 0.01). Variation in anthropometric measure is similar to MI group. Significant improvements were found between control group and MI group and control group and MI+ group in many anthropometric measures: e.g., BMI (*t* = 3.32 *p* < 0.01 and *t* = 3.84 *p* < 0.01, respectively). MI+ group had a higher consume of calories from PA than MI group (*f* = 5.24, *p* = 0.02).

*MI, motivational interviewing; BMI, body mass index; MWH, weight-for-height percentage; Fat%, fat percentage; BH, body height; BW, body weight; WC, waist circumference; HC, hip circumference; TSF, triceps skinfold thickness; Abd, abdominal thickness; SBP, systolic blood pressure; DBP, diastolic blood pressure; PA, physical activity.*

The total number of participants across the studies was 1820 children of which 44,4% were males and 55,6% were females with participants’ mean age equal to 6,21 years of age. Five interventions recruited participants through the health care system and one study through primary schools. One research included children aged between 8 and 11, all other works also involved pre-school aged children.

Overweight children were included in the sample population of each study except for one that solely involved obese children. Three studies excluded obese children from their sample. There is only one research involving normal-weight children on condition that one of its parents is obese.

MI is used as a stand-alone treatment in all analyzed studies. In all selected studies MI sessions were provided to parents of overweight and obese children, in one study the MI sessions were directed to children.

Four intervention used MI as it is described by Miller and Rollnick (2002), one applied the principles of brief MI (Dunn and Rollnick, 2003), only one study used a prevention protocol based on theories and models of behavioral change, i.e., the stages of change model, the attitude-social influence-efficacy model and MI techniques (Prochaska and DiClemente, 1992; Vries and Mudde, 1998; Miller and Rollnick, 2002).

### Results of Selected Studies

In Schwartz et al. (2007) mean BMI percentiles decreased in all three groups with differences reported between baseline and 6 months follow-ups: the control group decreased from 84.4 to 84.0, the minimal intervention group from 83.2 to 81.4 and the intensive intervention group from 81.8 to 78.7, although the reported differences were not statically significant. Significant within-group changes in eating behavior were found only in the minimal (decrease in intake of snacks) and intensive (decrease of dining out) groups. Ninety-four percent of parents reported to be very satisfied of the visit and they believed the intervention was beneficial to help them reviewing family’s eating habits.

In the High Five for Kids study (Taveras et al., 2011) the children’s intervention group had a small non-significant increase in the mean BMI after 1 year: children treated with usual care reported an increase of 0.49 (baseline: 19.1; after 1 year: 19.6), whereas children in the intervention group registered an increase of 0.31 (baseline: 19,2; after 1 year: 19,5). *Post hoc* analysis showed statistically significant effects on BMI in females (intervention group baseline: 19.2, after 1 year: 19.5; control group baseline: 19.3, after 1 year: 19.9) and in children living in families with lower income households (intervention group baseline: 19.6, after 1 year: 20.0; control group baseline: 19.9; after 1 year: 21.3), both belonging to the intervention group. Secondary outcomes revealed interesting results in terms of obesity-related behaviors: time spent watching video/TV greatly decreased in children belonging to the intervention group (from 2.67 to 2.13 h/day), fast food and sweet beverage intake reported a small decrease.

In Davoli et al. (2013) a difference in the variation of BMI scores 1 year after baseline between the control group and the intervention group has been found: in the control group the increase went from 18.21 to 19.00, whereas in the intervention group the increase was from 18.28 to 18.76. The interaction test revealed a strong interaction in the intervention group between the BMI variation and mothers with higher educational level, intended as more than 13 years of school attended (mean BMI score variation of -0.20 in intervention group and 0.84 in usual care), and a weaker effect among girls while no effect on boys. An increase in physical activity along with positive changes in eating habits has been reported in children assigned to the intervention group.

Children in the experimental group studied by Small et al. (2013) resulted having a smaller waist circumference and waist by height ratio, both after the intervention and until 3 and 6 months after. There were no statistically differences in the BMI percentile (intervention group baseline: 96.72, after the intervention: 93.74, 3 months post-intervention: 92.42, 6 months post-intervention: 95.13; control group baseline: 95.40, after the intervention: 94.74, 3 months post-intervention: 90.19, 6 months post-intervention: 94.74) among the two groups.

After a 2-years follow up van Grieken et al. (2013) registered a lower increase in BMIs in children assigned to the intervention group compared to the control group with children who were mildly overweight at baseline (baseline BMI 17.25 and 17.50, estimated adjusted difference -0.67 and -0.52, respectively). No significant differences in BMIs and waist circumference was found between groups after a 2-years follow up.

Wong and Cheng (2013) reported a significant pre-post intervention difference in all anthropometric measures for MI group except for body height, mean BMI decreased from 23.39 at baseline to 22.72 after 14 weeks. Similar significant differences in anthropometric measures mean values were found in MI+ group except for hip circumference and abdominal thicknesses, mean BMI decreased from 23.71 at baseline to 22.54 after 14 weeks. Children in the control group showed a significant deterioration in the anthropometric measures from the baseline to 14 weeks and mean BMI decreased from 23.86 at baseline to 24.67 after 14 weeks.

Significant improvements have been found comparing anthropometric measures between the control group and the MI and the MI+ group, respectively. Children belonging to MI+ group have reported a significant higher change in calories consumed compared with the MI group. A significant improvement in eating habits and an increase in physical activity with subsequent increase of calories consumed has also been reported for both experimental groups.

## Discussion

Among the studies reported so far in this review, three show that MI intervention is more effective than the usual care for changing BMI in children enrolled in obesity treatments.

Wong and Cheng’s (2013) study reports a statistically significant decrease of the BMI and of some anthropometric measures in all participants belonging to both intervention groups. Children in this study have been directly provided with MI sessions, allowing the clinicians to tailor the MI intervention according to the children’s change condition. The above condition might have supported the treatment efficacy and good results in terms of weight loss and response to motivational intervention were achieved. The study also reports the increment of physical activity in MI+ group, that might be related to the influence of parents who have been provided with MI telephone consultation. Taveras et al. (2011) and Davoli et al. (2013) pointed out a strong relationship between BMI variation and some children’s characteristics among those who were delivered the MI intervention. A statistically significant BMI variation at the end of treatment was found only on females, on participants belonging to low-income families (Taveras et al., 2011) and on children whose mother had a higher educational level (Davoli et al., 2013). It is important to note that a small increase of children’s BMIs after the intervention may still represent a positive result as this could be coherent with the increasing trend of BMI-for-age curves (Kuczmarski et al., 2002).

Relevant results have been reported on secondary behavioral outcomes. Statistically significant variations were found on food calories intake and calories consumption from physical exercise (Wong and Cheng, 2013), the decrease of time spent watching TV (Taveras et al., 2011), the reduction of sweet snacks and drinks consumption (Davoli et al., 2013). Parents reported to be satisfied with the MI intervention in Schwartz et al. (2007) and Taveras et al. (2011) studies, suggesting that MI might be a well-accepted intervention from parents.

The analysis presented in this review evidences some methodological issues that might have negatively influenced the results of the reported studies, such as non-randomization of patients or practitioners, small sample size, unbalanced sample characteristics, low attendance to intervention activities, the absence of MI training and supervision evidence.

The studies analyzed provide very few details regarding MI practitioners’ training and none of them included the proficiency of counselors who delivered the motivational intervention. Only Schwartz et al. (2007) reported that pediatricians and registered dietitians added to MI training an assessment of MI interventions through audiotape recording, supervision and feedback.

Considering the importance for each reviewed study on the assessment of MI interventions efficacy, it is essential to evaluate if the counselor was actually delivering an “MI treatment” (Forsberg et al., 2010). The Motivational Interviewing Treatment Integrity (MITI) scale has been developed to address the need of monitoring MI skills (Moyers et al., 2005). It is a cost-effective tool allowing the evaluation of MI proficiency, the pre-post training MI ability development and the integrity of MI trial clinic interventions. Adherence to MI possibly assessed with the use of MITI is a key-point necessary to compare the different methods and interventions.

## Future Directions and Conclusion

The collected and analyzed data show that MI may be a method applicable in the treatment of overweight and obese children in the pediatric setting. Three studies come out with positive results on BMI, eating habits and lifestyles change. Overall, the efficacy of MI in the treatment of obese children cannot be empirically evidenced so far, given the insufficient data and the paucity of studies carried out on the focused age-group.

Further research is needed to provide more and useful data to better demonstrate the efficacy of MI interventions overcoming the methodological issues formerly reported. New protocols assessing the effects of MI on overweight and obese children have already been published and results will be soon issued, for instance Resnicow et al. (2012) designed a cluster-randomized intervention involving a large sample size (663 children aged 2–8 years) and Bean et al. (2014) proposed a randomized controlled trial with the assessment of the adherence to MI interventions delivered using the MITI 3.1 scale.

It seems necessary to carry out researches aimed at clarifying the effects of MI interacting with the children’s characteristics (e.g., baseline BMI, gender, family education level, household income) to understand with whom MI works better and how it can promote behavior changes.

MI application on children has still some unanswered questions, mainly directed to investigate whether it is better to involve children alone and/or with his/her parents to promote lifestyle changes and how MI should be tailored to favor behaviors that may lead to a healthier weight-levels.

## Conflict of Interest Statement

The authors declare that the research was conducted in the absence of any commercial or financial relationships that could be construed as a potential conflict of interest.
